# IMPROVING RECOVERY FROM HOSPITALIZATION IN GERIATRIC PATIENTS THROUGH WHEY SUPPLEMENTATION AND TESTOSTERONE

**DOI:** 10.1093/geroni/igae098.3595

**Published:** 2024-12-31

**Authors:** Michelle Yang, Pratik Gongloor, Rachel Deer, Elena Volpi, Shuang Li, Erin Hommel

**Affiliations:** University of Texas Medical Branch, Galveston, Texas, United States; University of Texas Medical Branch, Galveston, Texas, United States; The University of Texas Medical Branch, Galveston, Texas, United States; University of Texas Health San Antonio, San Antonio, Texas, United States; University of Texas Medical Branch, Galveston, Texas, United States; University of Texas Medical Branch, Galveston, Texas, United States

## Abstract

**Background:**

Sarcopenia, the age-related loss of muscle mass and strength, remains a significant concern among hospitalized geriatric populations due to functional decline and increased mortality. A previous pilot study demonstrated potential benefits for testosterone or whey supplementation (WS) during and post-hospitalization towards improved physical function and reduced re-admissions. Our follow-up study assesses the feasibility and preliminary efficacy of these interventions.

**Methods:**

This Phase I, single center, randomized clinical trial enrolled 80 participants, 65 years+, hospitalized for acute conditions. Participants were randomly assigned to one of four interventions: placebo oral and injectable (PO/PI) supplement, PI-WS, PO-testosterone injection (TI), or WS-TI. WS was provided twice daily during and post-hospitalization. TI was provided once pre-discharge. Testing was conducted at baseline and 4-weeks post-hospitalization. Primary outcome included Short Physical Performance Battery (SPPB) score change. Secondary outcomes included readmission rate and additional measures of physical function and body composition.

**Results:**

Mean age was 71.5 years with 70% female. Age-adjusted Charlson Comorbidity Index was 4. Fifty-two participants (65%) completed the study; 15 were re-hospitalized and 13 withdrew or were lost to follow-up. Among those completing the 4-week intervention, we found no statistically significant differences in SPPB score or readmission rate.

**Conclusion:**

We failed to demonstrate significant improvements in recovery among geriatric patients receiving testosterone, WS, or combination therapy. Compared to the previous study, participants were younger, healthier, and less likely to complete follow-up. We need further studies with larger samples of frail older adults to elucidate the relationship between these interventions and post-hospital functional recovery.

